# Structure and solvents effects on the optical properties of sugar-derived carbon nanodots

**DOI:** 10.1038/s41598-018-25012-8

**Published:** 2018-04-26

**Authors:** Nikolaos Papaioannou, Adam Marinovic, Noriko Yoshizawa, Angela E. Goode, Michael Fay, Andrei Khlobystov, Maria-Magdalena Titirici, Andrei Sapelkin

**Affiliations:** 1 School of Physics and Astronomy, Queen Mary, University of London, 327 Mile End Road, London, E1 4NS UK; 2 Materials Research Institute, Queen Mary University of London, Mile End Road, E14NS London, UK; 3 School of Engineering and Materials Science, Queen Mary University of London, Mile End Road, E1 4NS London, UK; 4 Electron Microscope Facility, TIA, AIST, 16-1 Onogawa, Tsukuba, 305-8569 Japan; 5 Department of Materials, Faculty of Engineering, Imperial College London, London, SW7 2AZ UK; 6 Nanoscale and Microscale Research Centre, University of Nottingham, University Park, NG7 2RD Nottingham, UK; 7 School of Chemistry, University of Nottingham, University Park, Nottingham, NG7 2RD UK

## Abstract

Carbon nanodots are a new and intriguing class of fluorescent carbon nanomaterials and are considered a promising low cost, nontoxic alternative to traditional inorganic quantum dots in applications such as bioimaging, solar cells, photocatalysis, sensors and others. Despite the abundant available literature, a clear formation mechanism for carbon nanodots prepared hydrothermally from biomass precursors along with the origins of the light emission are still under debate. In this paper, we investigate the relationships between the chemical structure and optical properties of carbon nanodots prepared by the hydrothermal treatment of glucose. Our major finding is that the widely reported excitation-dependent emission originates from solvents used to suspend the as-prepared carbon nanodots, while emission from dry samples shows no excitation-dependence. Another important highlight is that the hydrothermal conversion of biomass-derivatives under subcritical conditions leads to a heterogeneous mixture of amorphous-like nanoparticles, carbon onion-type and crystalline carbons composed of at least three different phases. The potential chemical reaction pathways involved in the formation of these hydrothermal carbon products along with a comprehensive structural and optical characterization of these systems is also provided.

## Introduction

Carbon nanodots (CNDs) represent a new family of carbon nanomaterials. The term refers to nanoscale (usually around or below 10 nm in diameter) particles consisting of mostly carbon atoms and exhibiting photoluminescence (PL) without necessarily showing evidence of quantum effects^1^. CNDs are promising candidates to replace the toxic metal-based semiconductor quantum dots (e.g. CdS, CdSe, CdTe, PbS, etc.) in applications such as bioimaging^2–4^, sensitizers for solar cells^5^, multicolour patterning^6–8^ and ions detection^9–11^. Advantages of CNDs over classical semiconductor quantum dots (QDs) include low cytotoxicity, chemical inertness, excellent biocompatibility, tunable surface functionalities, versatile and simple synthesis routes.

CNDs synthesis is divided in two main approaches, including top-down (cutting from different pre-formed carbon structures)^12–14^ as well as bottom-up (starting with molecular precursors)^3,15,16^ methods that yield a diverse array of CNDs. Hydrothermal carbonization (HTC) of biomass-derivatives has been proposed by Titirici *et al*. as an alternative route to produce carbon materials under milder conditions^17^. Recently this has been adopted for the production of CNDs whereby the CNDs represent the nucleation clusters, which are formed before the growth into the final HTC nanostructured carbon material^2,18,19^. Significant research efforts have been carried out to elucidate the origins of light emission in these materials. The optical properties of hydrothermal carbon nanodots are complex compared to classical organic fluorophores as well as semiconductor quantum dots. They are highly dependent on the carbon dots structural features. Some reports attribute the photoluminescence (PL) in CNDs to band gap transitions of conjugated π-domains within a small crystalline core^20,21^, while others correlate emission to surface-localized states^22,23^. The most reported PL characteristic in hydrothermal CNDs is excitation-dependent emission, while the origin of this phenomenon remains under debate^24^. Even though a number of reports link excitation-dependent emission to the presence of various surface chromophores or quantum confinements due to size differences^25,26^, yet clear evidence is still missing. Recent publications suggest that emission in CNDs changes in different solvents. For example, Wu *et al*. reported that fluorescence emission in their CNDs synthesized via one-step pyrolytic route changed from 400 to 430 nm when suspended in different solvents under the same excitation wavelength^27^. Lin *et al*. indicated that CNDs synthesized from m-phenylenediamine showed strong solvent-dependent emission between 400–500 nm^28^. Sciortino and co-workers found a dependence of the emission energy and decay kinetics on solvent properties, which highlights the crucial role of surface moieties^29^. Reckmeier *et al*. showed that emission of edge and surface bands undergoes opposite solvatochromic shifts when solvent polarity is changed^30^. This phenomenon is very similar to the solvatochromism in organic dyes, which is usually attributed to the intramolecular charge transfer but is rarely reported in CNDs^31,32^. None of these reports has actually investigated the differences in PL between the particles in dry state and in solvents with different polarities. Furthermore, there has not been a clear correlation between PL and physicochemical features of CNDs (i.e. functional groups, crystallinity). A typical issue CNDs face in their solid state is very weak fluorescence due to the direct π-π interactions similar to organic molecules, which is caused by an excessive resonance energy transfer observed in dry state^33^. To overcome this, Chen and co-workers prepared CNDs and by controlling their surface or interparticle spacing obtained successfully for first time solid-state fluorescence (SSF)^34^.

Literature reports on the formation mechanism of hydrothermal CNDs are scarce. Hu *et al*. proposed a formation mechanism of CNDs prepared from citric acid and monoethanolamine including polymerization, aromatization, nucleation and growth. Initially, monoethanolamine reacts with citric acid through dehydration and polymer nanoparticles with large diameter are formed. Upon heating, the polymer nanoparticles shrink due to continuous dehydration and C=C and C=N bonds are formed. In parallel, aromatic clusters are formed inside the nanoparticles and when their concentration reaches the critical supersaturation point, nucleation of carbon dots takes place. Further nuclei are formed by enhanced aromatization of the polymers and over time carbon dots increase in both number and size. Finally, the polymers disappear and carbon dots remain^35^. Similar approach was reported by De *et al*. for the formation of their CNDs under pyrolysis^36^. Similarly, Sun *et al*., reported the formation mechanism of CD from citric acid and urea under hydrothermal conditions, which promote formation of amide between -NH_2_ and -COOH followed by intramolecular hydrolysis between neighbour amide and COOH groups leading to formation of pyrrolic N within the carbon framework^37^. Xiao *et al*. used a polymer (PEG400) in presence of NaOH for the synthesis of CNDs. Reaction occurred when the carbon source pyrolyzed and oxidized in the alkaline environment which resulted in the formation of small particles that progressively aggregated to form clusters and finally the CNDs^38^.

Hydrothermal carbonization of biomass derivatives such as glucose utilized here is a complex process. Our group has previously studied the formation mechanism of amorphous carbon microspheres from glucose forming via a nucleation-polymerization process. (See Supplementary Information, Scheme S1)^39^.

Herein, we examine the relationship between structural and optical properties in CNDs prepared by HTC from glucose selected as a model system and examine their PL in different environments including the dry state to examine thoroughly the solvatochromic phenomenon. Furthermore, we propose a mechanism of formation of CNDs during HTC synthesis process.

## Results and Discussion

**Figure 1:**
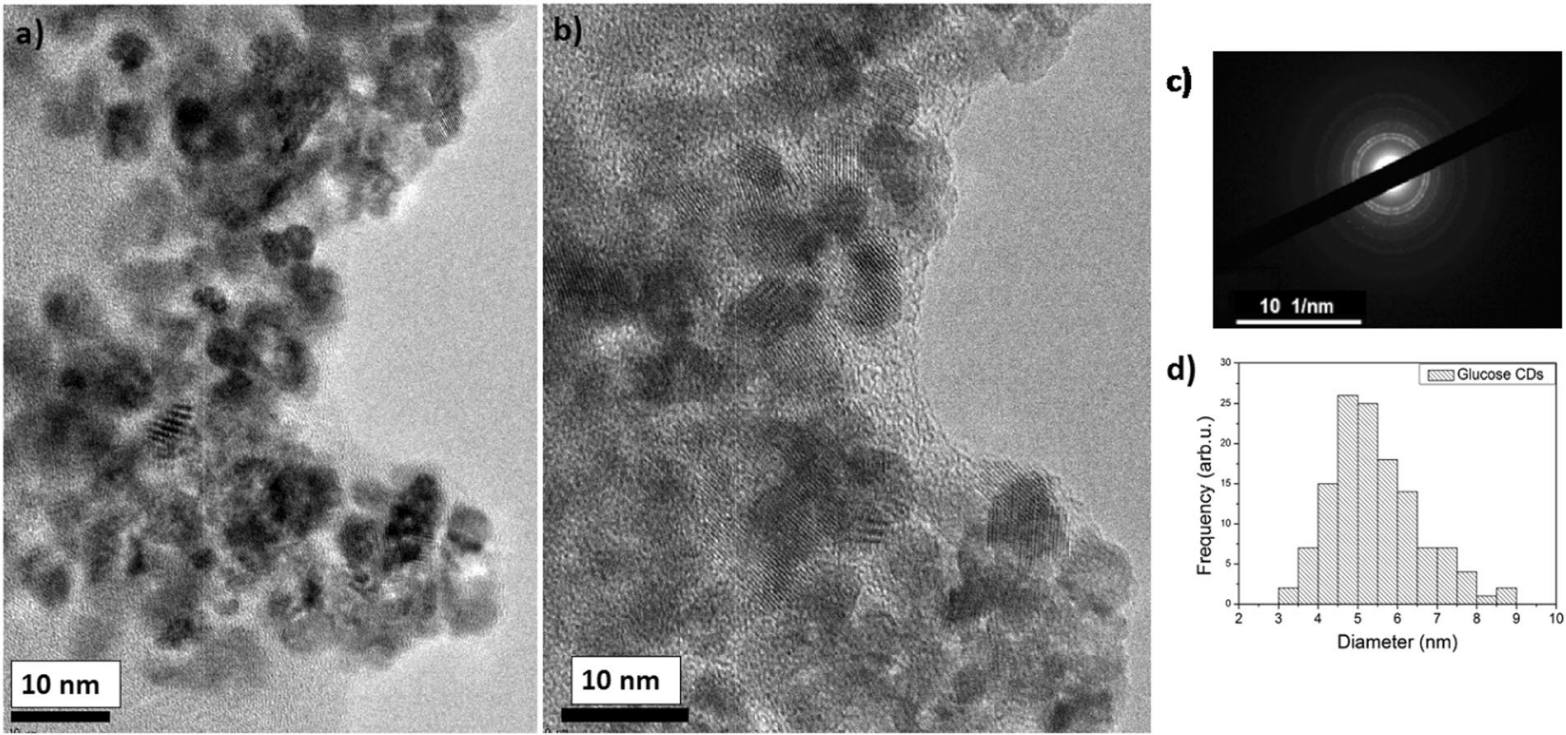
(**a** and **b**) TEM of glucose derived CNDs taken at 200 kV showing crystalline nanoparticles, with diameters below 10 nm, surrounded by an amorphous carbon matrix; (**c**) electron diffraction pattern of (**a** and **b**,**c**) average particle size distribution of the particles in (**a** and **b**).

CNDs were prepared via hydrothermal carbonization (HTC) of glucose by simply treating a 4% w/v aqueous glucose solution at 200 °C for 6 hours followed by centrifugation and filtration to isolate the CNDs from the larger micrometer sized carbon particles. As-prepared CNDs appear as yellow aqueous suspension that shows strong blue emission when illuminated by UV light (see Fig. S1). This is in good agreement with previous reports where many other carbon-based precursors were used^40–43^. Further, the glucose-derived CNDs solution was freeze dried to obtain a powdered form of the product. Initially, we investigated the morphology of the resulting glucose-derived CNDs from aqueous suspension using transmission electron microscopy (TEM) at 200 kV. (Fig. 1a,b).

The TEM images show crystalline CNDs where clear lattice fringes are observed which are surrounded by an amorphous carbon matrix. The electron diffraction patterns (Fig. 1c) confirm the crystallinity of our glucose-derived CNDs showing lattice fringes spaced around 0.32 nm, which is close to the graphite (002) (0.3354 nm). The size of the carbon nanodots was measured from TEM images using ImageJ software, and histograms of the size distributions are shown in Fig. 1d from which the average size and standard deviation was calculated. Based on the statistics of about 120 particles in several TEM images, size distribution measurements show that the average diameter of the as synthesized glucose carbon nanodots is (5.15 ± 0.83) nm.

**Figure 2:**
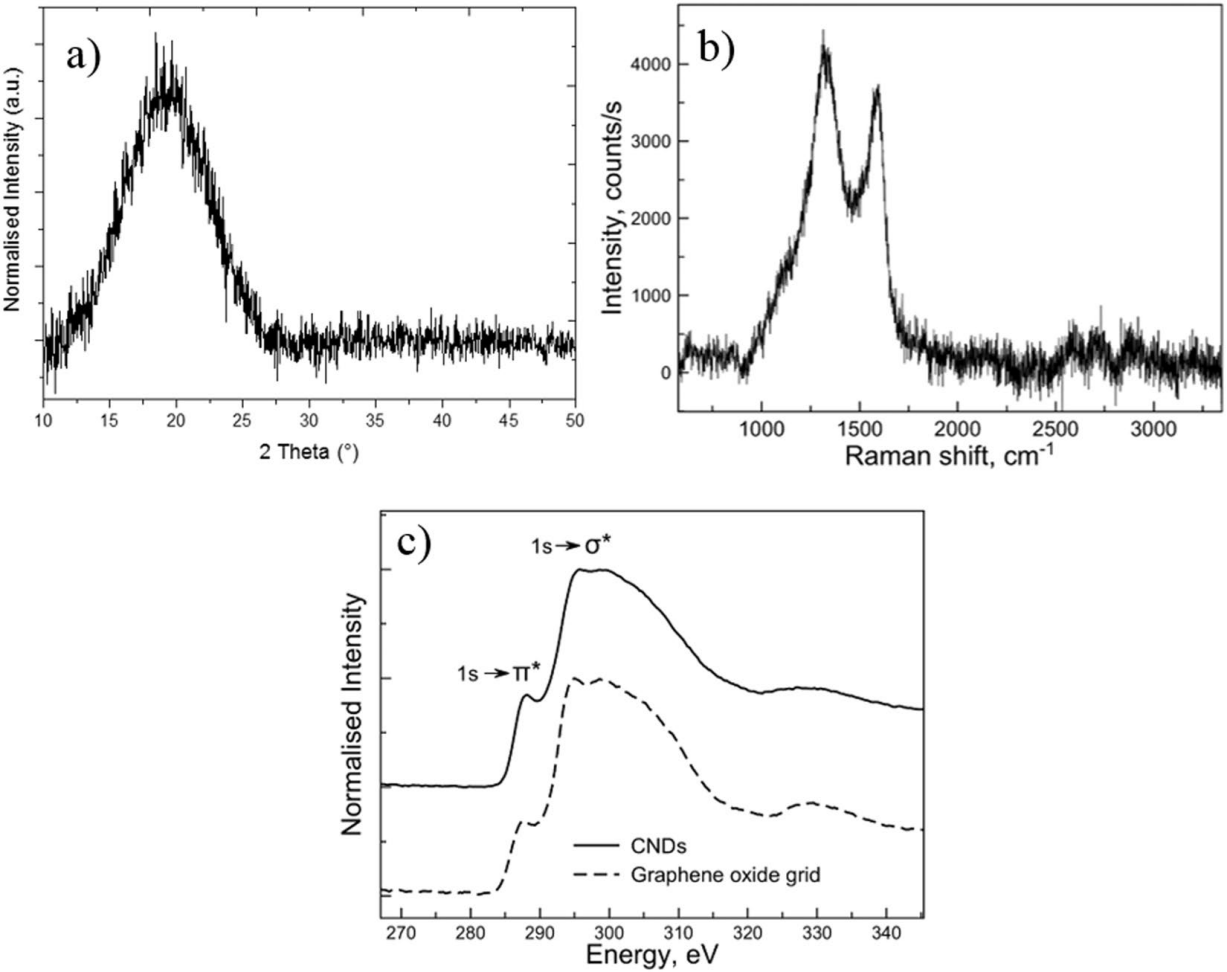
Raman (**a**), XRD (**b**) and EELS (**c**) data for glucose CNDs together with the graphene oxide grid reference.

Our XRD and Raman data recorded on freeze-dried CNDs (Fig. 2) do not fully support the presence of crystalline nanoparticles. The XRD pattern shown in Fig. 2a shows a single broad peak at around 22.5° again indicating a significant level of topological disorder in the sample. The 002 peak at about 2θ = 22.5° corresponds to a set of sp^2^ carbons-graphitic carbons with stacking faults, known as turbostratic carbons. No other peaks are detected in XRD suggesting amorphous nature of our materials.

Raman data are shown in Fig. 2b, while the results from Raman analysis are summarized in Table 1. We clearly observe two of the main Raman peaks normally found in low dimensional carbon systems (e.g. carbon nanotubes, graphene, graphene oxide, carbon black): D and G bands around 1330 cm^−1^ and 1590 cm^−1^ respectively. Note that D band is not observed in perfect crystalline graphite nor graphene and it is usually associated with some form of lattice disorder (e.g. impurities, cluster size effects, presence of sp^3^ bonds etc.^44^). Combinations of Lorentzian, Gaussian and Breit-Wigner-Fano (BWF) peaks are normally used to extract numerical information^45^. Considering that G band in all samples is clearly asymmetric we used Lorentzian peak shape to fit D band and BWF shape to fit G band. We also tested a combination of double Gaussian fitting and Gaussian + BWF, but neither provided an adequate fit to the data.

**Table 1:** Summary of the analysis of Raman data for all carbon nanodots.

	*D* band, Lorentzian [cm^−1^] Position/FWHM	*G* band, BWF [cm^−1^] Position/FWHM
Graphene	—	1586/10^73^
Graphite	—	1582/12^74^
Glucose nanodots (size: 5.2 nm)	1322/224	1584/102

Data fitting has been carried out using Lorentzian peaks. The last column shows the ratio of *D* to *G* band intensity.

The Raman data of CNDs indicate that the sample has a significant degree of disorder which is reflected in increased D and G peaks width^45^. D band originates from aromatic conjugated sp^2^ six-membered carbon rings^46^, hence an increase in D peak intensity reflects large number of such ring clusters. Consequently, broadening of D band reflects disorder in aromatic clusters. Looking at D band peak width information in Table 1, we observe that glucose-derived CNDs show a relatively high level of aromatic ring disorder. The position of the D band strongly depends on excitation energy^47^ and for He-Ne (632.8 nm) laser used here the peak is expected at around 1325 cm^−1^ in graphite. This position is close to the ones observed in our sample, suggesting presence of graphitic structures in our samples. Raman signal (albeit weak) in the region between 2500 cm^−1^ and 3000 cm^−1^ provides further evidence for presence of graphite-like structures (see Fig. 1a), as opposed to simply amorphous carbon. Single layer graphene exhibits very strong and narrow 2D peak at around 2700 cm^−1 ^^48^, which is subject to broadening, splitting and reduction of intensity in multi-layered graphite. In our case, the signal in this region is weak, suggesting the presence of disordered multi-layered graphite-like structures.

The observed G band is clearly asymmetric which, on the lower wavelength side of the peak in nanoscale systems, is usually associated with the phonon confinement^49^, thus suggesting that the particle size is sufficiently small for bulk phonon dispersion description to break down. It is worth pointing out, that our glucose CNDs samples show Raman signal very similar to that observed in graphene oxide^50–53^, which may suggest significant contribution of out-of-plane bonding.

The EELS at carbon K edge shows a broad peak at around 285 eV corresponding to 1s-π* transition observed in *sp*^2^ sites in graphite^54^, followed by a broad 1s-σ* peak at around 300 eV corresponding to both *sp*^2^ and *sp*^3^ sites^55^. The weak exciton peak at the onset of the 1s-σ* feature is not observed in amorphous carbon, and further indicates the presence of graphitic bonding^56^. The EELS signal is very similar to that of the graphene oxide reference (Fig. 2c) indicating contribution from both *sp*^2^ and *sp*^3^ bonded carbon. Further comparison with the published EELS^57–59^ data allows placing our samples closer to graphene oxide-amorphous carbon side of the amorphous carbon-graphene oxide-graphene sequence.

These findings suggest that although the XRD pattern in Fig. 2a shows a level of disorder similar to that found in amorphous carbons, Raman exhibits sensitivity to significantly more ordered structures. At the same time, EELS yields data that agree with both the XRD and Raman results, suggesting the existence of predominantly amorphous carbon features with small amounts of graphitized samples and crystalline *sp*^3^ nanodiamond-like structures.

**Figure 3:**
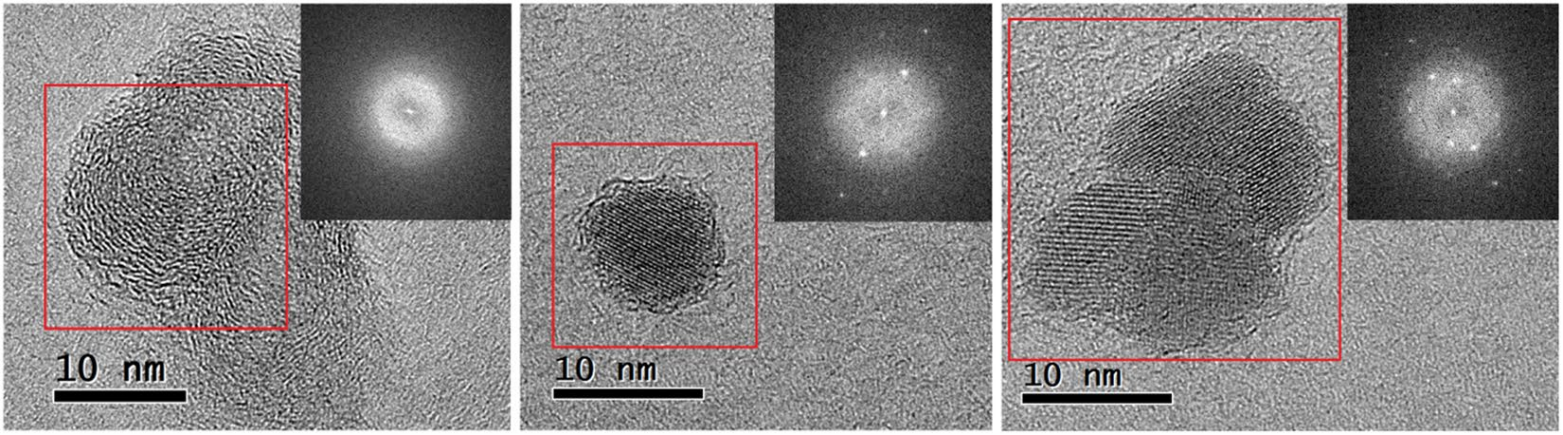
TEM micrographs of the glucose-derived CNDs taken at 80 kV showing a number of different crystalline structures: amorphous structure (**a**), onion-like carbon structure and amorphous crystalline structure (**b**) crystalline carbon and (**c**) a mixture of expanded graphite and crystalline carbon. Fast Fourier transforms taken from the red-boxed areas are inset.

In order to further clarify the crystalline structure of the CNDs derived from the hydrothermal carbonization of glucose and to reduce the rate of knock-on damage under illumination by 200 kV electrons, we have acquired HRTEM images at a lower operating voltage of 80 kV. These TEM micrographs are shown in Fig. 3. Even at 80 kV, we observed beam-induced sample crystallization at a dose of 2000 electrons/Å^2^ over a 30-minute timeframe (see Supplementary AVI file data taken at 0.5, 1, and 5 minute intervals). Major changes can be seen after about two minutes of beam exposure. Therefore, all our TEM images at 80 kV have been acquired well within this 2-minute timeframe.

The 80 kV HRTEM images and corresponding fast Fourier transforms in Fig. 3a–c show a mixture of at least three types of particles: carbon-onion type, crystalline carbon and graphitic like carbon. Fig. 3a shows the typical pattern of carbon onions with distances between neighbouring shells of about 3.5 Å. Fig. 3b,c shows a crystalline structure with an interatomic distances of 2.23, 2.33, 2.5 and 2.8 Å suggesting a mixture of *sp*^2^ and *sp*^3^ bonded structures possibly covered by an amorphous carbon layer - an effect previously reported for nanodiamonds^60,61^. We also observe graphitic-like interatomic distance of 3.7 Å corresponding to the (002) of expanded graphite (Fig. 3c). Unlike with the majority of the TEM studies reported in the literature, we observed a heterogeneous structural profile of CNDs, which are subject to further examination in order to understand their exact characteristics.

**Figure 4:**
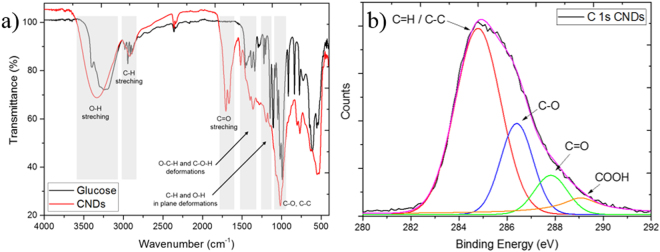
FTIR (**a**) and XPS (**b**) spectra of glucose-derived CNDs.

FTIR and C 1 s XPS spectra show indeed the presence of various oxygenated groups on the surface of glucose CNDs. FTIR spectra of glucose-derived carbon nanodots (Fig. 4a) shows the presence of O-H stretching vibrations (3600–3200 cm^−1^) along with C=O stretching vibrations at 1695 cm^−1^. A combination of O-C-H and C-O-H absorption bands occur in the region of 1525–1350 cm^−1^. In plane C-H and O-H deformation can be observed at 1170 cm^−1^ and a band of C-O and C-C stretching was assigned from 1191 cm^−1^ to 995 cm^−1^. The bands in the region of 875–750 cm^−1^ are assigned to aromatic C-H out of plane bending vibrations^62,63^. The presence of O-H groups and C-O-C linkages were also confirmed in the deconvoluted C 1 s spectra of glucose nanodots with the peak at 286.4 eV (Fig. 4b,c and Table S1 for more detailed results).

It is not very clear whether the oxygenated functional groups are directly linked to the surface of the crystalline carbon nanocrystals or if they are located on the surface of the amorphous carbon matrix surrounding each individual fluorescent carbon nanocrystal. Presumably there is a combination of both which explains the expanded graphitic structure observed from electron diffraction (3.7 nm). Nevertheless, XPS and FTIR show that glucose-derived NDs have a high number of polar oxygenated functionalities on their surface consisting of a mixture of hydroxyl, carboxyl and carbonyl functionalities.

**Figure 5:**
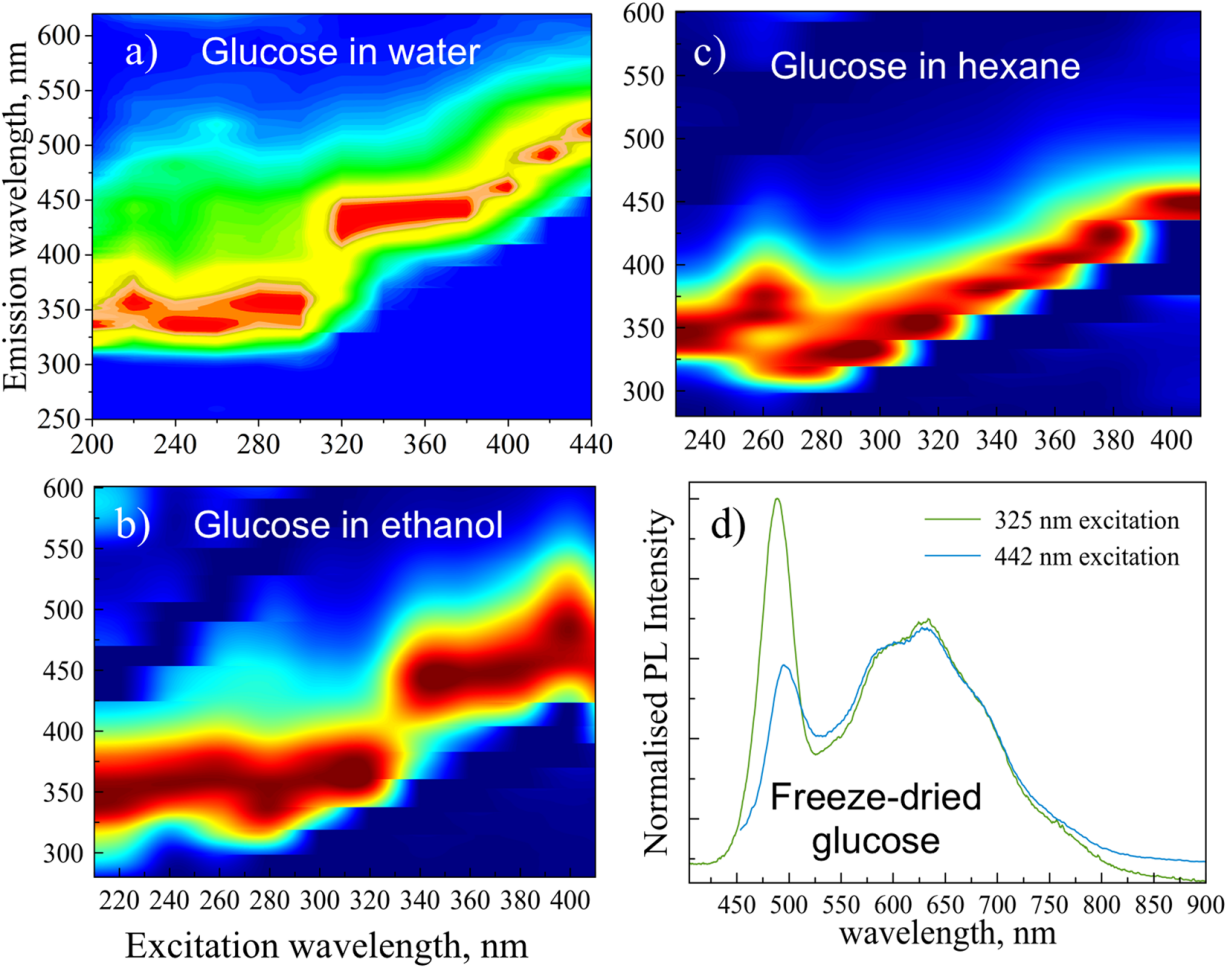
PL maps glucose CNDs in water (**a**, relative polarity 1); ethanol (**b**, relative polarity 0.654) and in hexane (**c**, relative polarity 0.009). Normalized PL data of freeze-dried glucose sample with two excitation wavelengths (325 nm and 442 nm) are also shown (**d**).

Optical properties of CNDs have attracted significant interest with the vast majority of the reports on light emission in CNDs synthesized by HTC route indicate excitation-dependence of the light emission. The detailed excitation-dependent photoluminescence experiments reveal a complex picture of light emission (Fig. S2B and C) with strong emission in the blue-green region of the visible light spectrum. These emission spectra show a change in the shape of the spectra together with the excitation-dependent shift usually observed in photoluminescence (PL) spectra of CNDs suspended in water. This change in the shape is consistent with the structural variations of glucose-derived CNDs observed in TEM (see Fig. 3), indicating an interplay between contribution of various structures seen in TEM to the light emission. Due to the observed complexity of the PL spectral shapes and their excitation-dependence, we produced PL maps to further understand light emission in these systems. From the photoluminescence map (recorded in water suspension) for glucose-derived CNDs (Fig. 5a), we can see that PL spectra show clear dependence on the excitation wavelength, however one can clearly see that this dependence of the PL in water is due to several distinct regions (i.e. 200–290 nm; 290–360 nm and 360–440 nm, see Fig. 5a) where emission is in fact nearly excitation independent, clearly pointing to (and potentially allowing to identify) discrete transition between different emission channels as the excitation wavelength is changed.

The majority of the PL data on CNDs prepared by HTC reported in the literature so far are collected in a colloidal water suspension^16^ or in some other solvent, such as ethanol^64^ or acetic acid^65^. Solvent polarity is likely to have a strong effect on the surface-related light emission and it has been reported recently^66^ that pH can have noticeable effect on the light emission. Thus, in order to further examine origins of the observed excitation-dependent light emission we have collected maps of glucose-derived CNDs (see Fig. 5a–c) in water (relative polarity 1), ethanol (relative polarity 0.654) and hexane (relative polarity 0.009). These data show clear effects of sample-solvent interaction as maps are changing noticeably both above and below 320 nm excitation. In addition to influencing emission, these solvents seem to also noticeably affect the absorption signal in glucose CNDs. (see Fig. S2A). The latter can, of course, reflect the differences in the solubility of the CND subtypes observed in the TEM (Fig. 3). More generally, we have observed strong absorption around 300 nm, which can correspond to the HOMO-LUMO gap, yielding an energy value of the gap of around 4.1 eV. Such value of the HOMO-LUMO gap corresponds to an aromatic structure with only 2–4 rings^67^.

In order to minimize the effect of solvents, the CNDs were freeze-dried and PL was measured using 325 nm and 442 nm laser excitation. Visually, the samples appeared as brownish-layered structures (Fig. S2D) and show bright white light emission (when illuminated by 325 nm laser light) contrary to the blue glow observed in a suspension. The normalized PL data for glucose are shown in Fig. 5d and one can see a significant difference between light emission in suspension and in dry samples. The bright white light emission in dry samples is clearly due to significant portion of the signal coming from the blue to red region of the spectrum. It is believed that the high abundancy in hydroxylic groups on the surface of CNDs, as revealed by the XPS and FTIR studies, assisted in preventing the aggregation induced quenching (AIQ) effect. Furthermore, the peak emission wavelengths shows no dependence on the excitation wavelength. Therefore, we conclude that vast majority of the PL reported to-date for HTC prepared CNDs could be due to particle-solvent interaction, rather than due to CNDs themselves. This conclusion is further corroborated by significant spectral changes (e.g. a narrow PL peak at around 500 nm).

The complex light emission observed in the colloidal suspension of CNDs is in good agreement with the heterogeneous mixture of several types of crystalline carbon phases detected by TEM (Fig. 3) and the diverse nature of functional groups revealed by FTIR and XPS. However, considering the result of TEM analysis, it is rather puzzling that such mild hydrothermal carbonization conditions (200 °C, 30 bars of self-generated pressure) lead to the formation of such defined crystalline structures. As mentioned in the introduction, our group has previously studied the formation mechanism of amorphous hydrothermal carbon spheres and an explanation on the formation mechanism of amorphous HTC spheres in provided in Supplementary Information. Briefly, these form from hydroxymethylfurfural (HMF) which results upon dehydration of glucose via the fructose isomerization. Subsequently HMF polymerizes into small (few nm in size) polymeric nanoclusters, which start growing up to few micrometers in size, and by further dehydration are transformed into hydrothermal carbonaceous spheres^17,68^.

The question arising is how the CND form during this process simultaneous with the HTC microspheres and why they have a crystalline heterogeneous nature. We believe that some of the polymeric nuclei formed initially during the hydrothermal process act as hot spots (due to temperature gradients existing in the autoclave during the HTC process). At the same time, CO/CO_2_ gas along with H_2_, are formed *in situ* in the autoclave from partial gasification of glucose under subcritical conditions. These gases deposit onto the hot spot nuclei in a CVD-like process via CO_2_ reduction under autogenic pressures (30 bars) leading to the formation of crystalline carbons. We hypothesize that the first crystalline form of carbon CNDs formed under such hydrothermal conditions is *sp*^3^-bonded carbon obtained from amorphous/carbon black type which then can further convert into carbon onions which subsequently form graphitic structures^69^. During incipient stage of glucose transformation via the “hot spots CVD” mechanism mentioned above, a mixture of *sp*^2^*/sp*^3^ carbon nanostructures are initially formed which are later converted into predominately *sp*^2^ carbon nanostructures^69^. It was also demonstrated^70,71^ that the intershell distance in carbon onions from nanodiamond depends on the temperature and pressure, varying from 3.2–3.8 nm. The transformation of nanodiamond into carbon onions has been demonstrated to be reversible under high temperatures and pressures^70,72^. The nanodiamond derived carbon onion further converts into nanographite (graphitic CNDs) through further annealing^71^. Due to the hydrothermal conditions the resulting crystalline structures are always accompanied by oxygenated functional groups, which explains the expanded graphitic structures. In addition, these crystalline phases in equilibrium are always accompanied by a larger amount of amorphous or polymeric like carbon resulting from HMF polymer clusters aggregation.

## Experimental Section

Chemicals: D-(+)-Glucose was purchased from Sigma-Aldrich. Deionized water was used for all the experiments.

Synthesis Procedure: D-(+)-Glucose was used as precursors to synthesize the HTC carbon materials. 4% w/v aqueous glucose solution and placed in a Teflon lined, stainless steel autoclave, which underwent hydrothermal treatment at 200 °C for 6 hours. The obtained dark brown liquid phase was centrifuged at 20,000 rpm for 10 min to separate the liquid containing fluorescent carbon nanodots from the solid black precipitate. The liquid phase containing carbon nanodots was then filtered using standard syringe filters. Further, the liquid phase was freeze dried to obtain the final powder of carbon nanodots which was used for further characterization.

Structural Characterization: Transmission electron microscopy (TEM) was performed on JEOL JEM-2010 electron microscope operating at 200 kV to observe the morphologies of the samples. Carbon nanodots were dispersed in ethanol and one drop was applied onto the copper grids coated with amorphous carbon for TEM measurements. HRTEM was performed in an image-corrected FEI Titan (S)TEM operated at 80 kV in order to reduce the effects of “*in situ*” graphitization. Electron energy loss spectroscopy (EELS) was performed on JEOL 2100 F FEG-TEM operating at 100 kV equipped with a Gatan Tridiem Filter. Raman spectra were measured using a Renishaw 1000 microspectrometer with excitation wavelength of 633 nm. X-ray diffraction (XRD) patterns were recorded using Panalytical Xpert Pro diffractometer equipped with Ni filtered Cu Kα radiation (λ1 = 1.5406 Å and λ2 = 1.5444 Å) collected in the 2θ range from 5° to 70°. Fourier Transform Infrared Spectroscopy (FTIR) measurements were recorded using a Bruker Tensor 27 instrument equipped with diamond lens Attenuated Total Reflectance (ATR) module in the range from 4000 cm^−1^ to 400 cm^−1^. XPS measurements were carried out on a K-Alpha spectrometer utilizing a monochromatic AlKα X-ray source (1486.6 eV, 400 μm spot size, 36 W).

Optical Characterization: The absorption spectra were recorded using the Perkin Elmer Lambda LS 35 UV-visible spectrometer. The fluorescence spectra were recorded using Perkin Elmer LS 55 fluorescence spectrometer. Slit width of 6 nm both for excitation and emission was used when measuring fluorescence emission of aqueous solution of carbon nanodots. The excitation wavelength was increased from 200 nm to 480 nm in 20 nm increments, and the corresponding fluorescence emission spectrum was recorded. Quartz cuvettes (Thor Labs) were used for the measurements of UV-Vis absorption spectra and fluorescence emission spectra. These quartz cuvettes have a 10 mm transmitted path length through the sample and a certified wavelength range from 200–3000 nm. We have chosen quartz cuvettes for UV-Vis absorption and fluorescence emission measurements because of their very good transmission spectra all the way down to 200 nm (78% transmission at 200 nm), while plastic and glass cuvettes show very low transmission below 290 nm and may contribute to the background emission.

## Conclusion

In this paper, we examined the structure and optical properties of CNDs produced from glucose by hydrothermal carbonization. We found that the product of the reaction is a heterogeneous mixture of amorphous-like, carbon-black type and various crystalline nanoparticles including carbon onions and expanded nanographite. We show that even at relatively low electron doses, effects of sample annealing by TEM are observed. We suggested a formation mechanism for these crystalline particles forming under such mild conditions based on HMF polymerization into HTC nuclei followed by formation of hot spots due to temperature gradients and deposition of “*in situ* formed” gases onto these hot spots under pressurized conditions leading to the formation of crystalline particles.

We have further examined light absorption and emission in these systems and found complex excitation-depended light emission for samples suspended in solvents. We found that this complex emission picture is clearly affected by the polarity of the solvent used to prepare colloidal suspension. Furthermore, we found that solvent-free samples show light emission drastically different from samples in colloidal solution and demonstrate no excitation- dependent light emission.

Thus, this work clearly points to the need for untangling effects of sample phase heterogeneity and surface functionalization on the light emission in HTC-prepared CNDs. This work suggests that future research in HTC-prepared CNDs must focus on resolving the problem of heterogeneity of the final products and ensuring adequate structural characterization using low dose TEM. Further support on the mechanism of formation should be achieved by performing a series of *in situ* measurements based on XRD (both WAXS and SAXS) and neutron scattering studies.

## Electronic supplementary material

Supplementary Information

Beam-induced sample crystallization
