# Supplementary material for: Structure and solvents effects on the optical properties of sugar-derived carbon nanodots
Source: Sci Rep. 2018 Apr 26;8:6559. doi: 10.1038/s41598-018-25012-8 (PMC5920085; doi:10.1038/s41598-018-25012-8)
Supplement: Supplementary file 1 — Supplementary Information [file 41598_2018_25012_MOESM1_ESM.pdf]

Supplementary Information

Structure and solvents effects on the optical properties of sugar-derived carbon nanodots

Nikolaos Papaioannou[1, 2], Adam Marinovic[3], Noriko Yoshizawa[4] Angela E. Goode[5], Michael Fay[6], Andrei Khlobystov[6, 7], Maria-Magdalena Titirici[2, 3]* and Andrei Sapelkin[1, 2]*

1. School of Physics and Astronomy, Queen Mary, University of London, 327 Mile End Road, London, E1 4NS, UK
2. Materials Research Institute, Queen Mary University of London, Mile End Road, E14NS, London, UK
3. School of Engineering and Materials Science, Queen Mary University of London, Mile End Road, E1 4NS, London, UK
4. Electron Microscope Facility, TIA, AIST, 16-1 Onogawa, Tsukuba 305-8569, Japan
5. Department of Materials, Faculty of Engineering, Imperial College London, London SW7 2AZ, UK
6. Nanoscale and Microscale Research Centre, University of Nottingham, University Park, NG7 2RD, UK
7. School of Chemistry, University of Nottingham, University Park, Nottingham NG7 2RD, UK

*Corresponding authors: m.m.titirici@qmul.ac.uk; a.sapelkin@qmul.ac.uk

Experimental results

Detailed XPS results of C 1s spectra are shown in **Table S1**.

Table S1: Summarised results of C 1s spectra of as-synthesized carbon nanodots.

**Name**	**C=C / C-C**	**C-O**	**C=O**	**COOH**
Glucose nanodots	60.2 %	21 %	9.1 %	9.7 %

The CNDs aqueous suspension under UV light illumination.

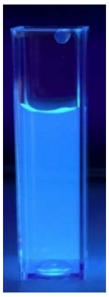

Figure S1. Light emission from glucose carbon nanodots in aqueous suspension under UV light.

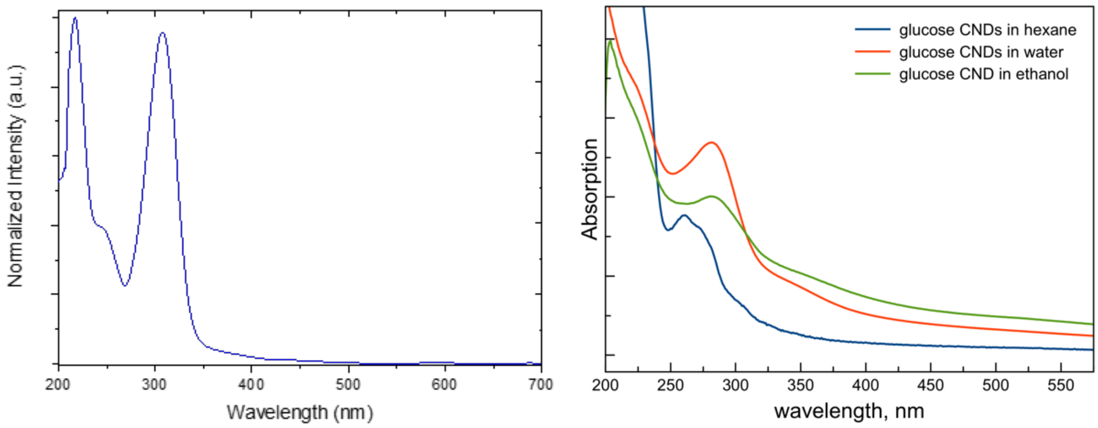

Figure S2A. Left: Light absorption in glucose carbon nanodots in aqueous suspension. Right: Light absorption in glucose CNDs suspended in ethanol and hexane.

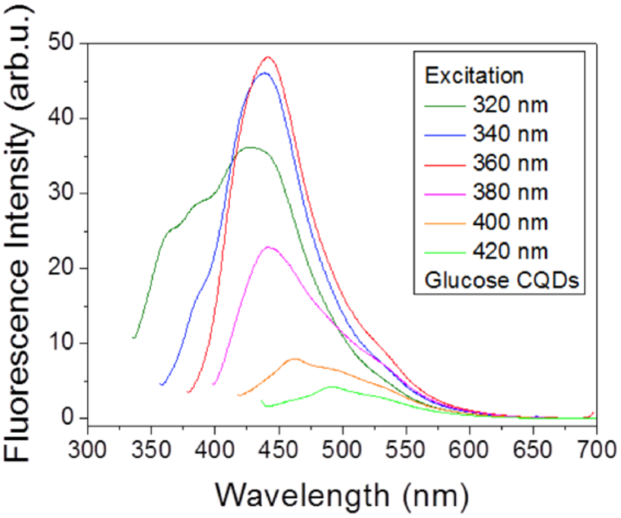

Figure S2B. Light emission spectra collected in aqueous suspension glucose carbon nanodots. These data were used in construction of the light emission maps.

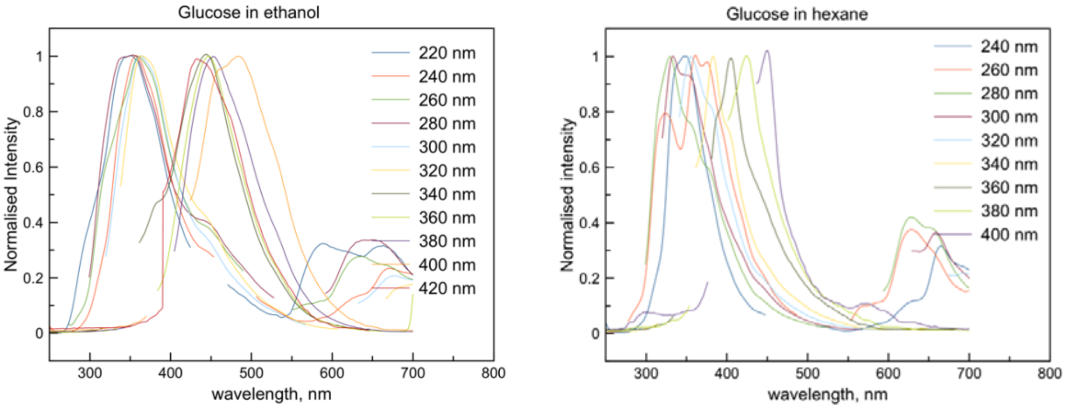

Figure S2C. Light emission spectra of glucose nanodots collected in ethanol (left) and hexane (right). These data were used in construction of the light emission maps.

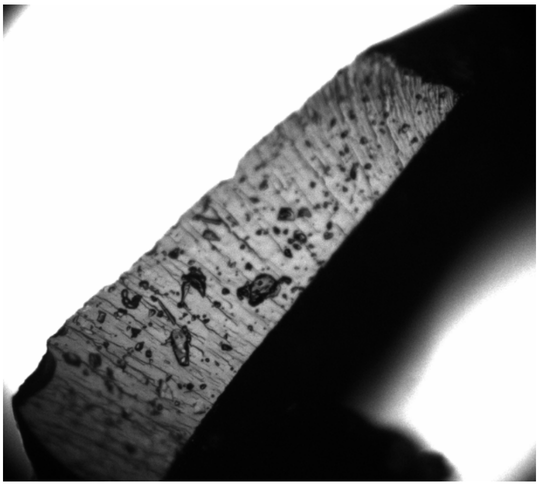

Figure S2D. Dark filed image of glucose sample freeze-dried powder flake at 20X.

Proposed reaction routes

During the hydrothermal carbonisation under subcritical regime, glucose is first isomerised to fructose followed by the elimination of three water molecules to form hydroxymethylfurfural (HMF). Chemically, HMF can be hydrolysed to further produce levulinic acid (4-oxopentanoic acid) (LA) and formic acid in a 1:1 ratio. (**Scheme S1**).[1]

HMF, which is in equilibrium with LA in water, can also undergo further complex reactions with the other products resulting in the formation of new carbon-carbon bonds.

HMF can for example undergo a well-documented condensation reaction of aromatic aldehydes known under the name of benzoin condensation. Self-benzoin condensation of HMF forms a coupled product called furoin (**Scheme S1b**).

Another possible reaction route for linking two or more HMF units together by C–C bond formation is the Diels–Alder reaction. A typical Diels–Alder reaction is a 4πs + 2πs reaction involving a diene and a dienophile in which all atoms participating in the reaction are unsaturated carbons[2] (**Scheme S1c**). Interestingly, the Diels–Alder reaction also ensures rate acceleration and enhanced selectivity in aqueous solution at elevated temperatures, which are the exact conditions in our system. This is attributed to the combined effects of hydrogen bonding of the water and enforced hydrophobic interactions. Successive condensations of more than two units of HMF are also possible giving six- and five carbon chains.

HMF can also undergo cross condensation with acetone and other aldehydes and ketones with at least an acidic α-proton known to be also formed during the glucose hydrothermal treatment degradation reaction (**Scheme S1d**). The cross-aldol condensation route has been demonstrated by Dumesic and co-workers.[3]

Along with the formation of these new C-C bonds, a growth in the molecular weight of the forming products occurs, and therefore new "carbonaceous" species are formed within the aqueous phase. This is when the nucleation occurs as a new phase has formed leading to the thermodynamic instability. The small nuclei formed at this stage and their size can be controlled by time and precursor concentration.

Obviously, the formation of the carbonaceous materials via HTC is a very complex process. The reactions provided in **Scheme 1** and **Scheme 2** are by far a simplified version of what is actually happening inside the reactor during the HTC process and should only be used as a tentative guidance. Similarly, the HTC chemical structure is also very complicated, containing sp^2 and sp^3 carbon atoms, various oxygenated functional groups (hydroxyl, lactones, ketones, aldehydes, carboxylic groups).

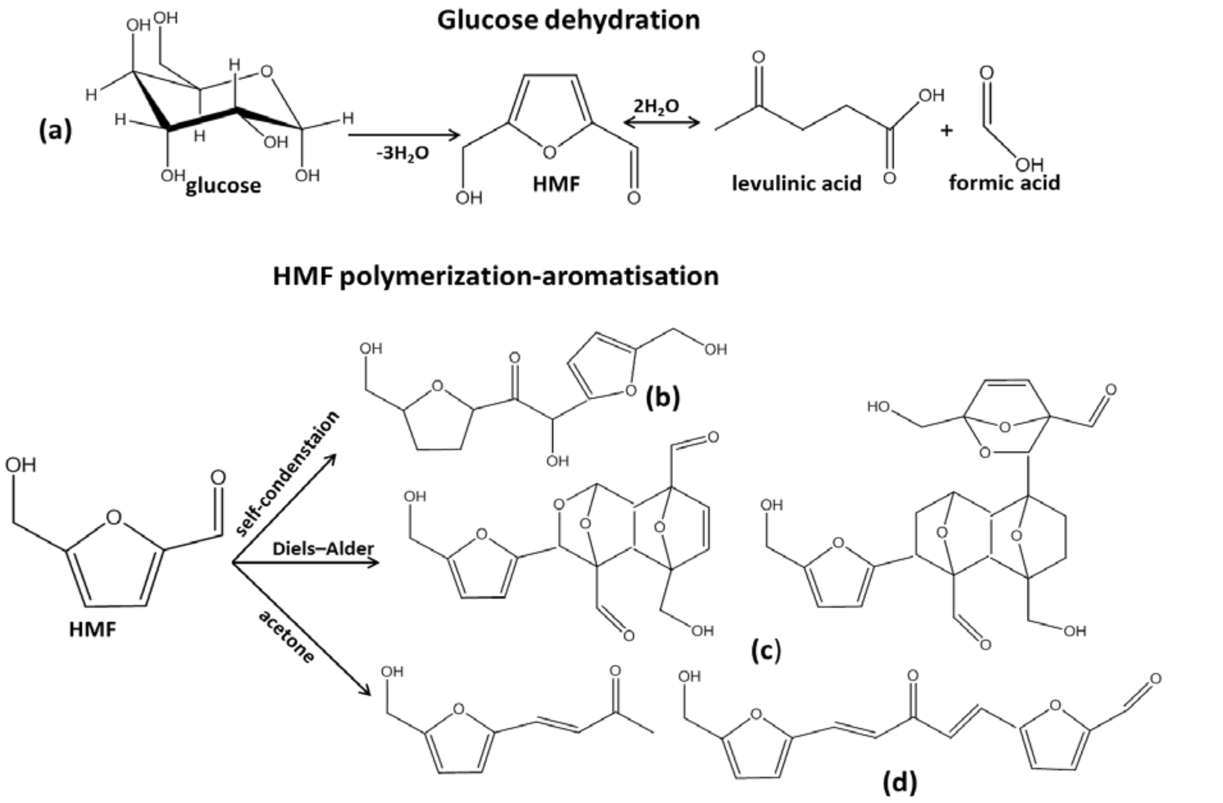

Scheme S1: Chemical reactions involved in the formation of carbon nanodots formed upon hydrothermal carbonization of glucose: a) Dehydration of glucose to HMF and potentially to levulinic and formic acid; b) Self-condensation of HMF; c) Diels Alder HMF condensations; d) Reaction of acetone with HMF

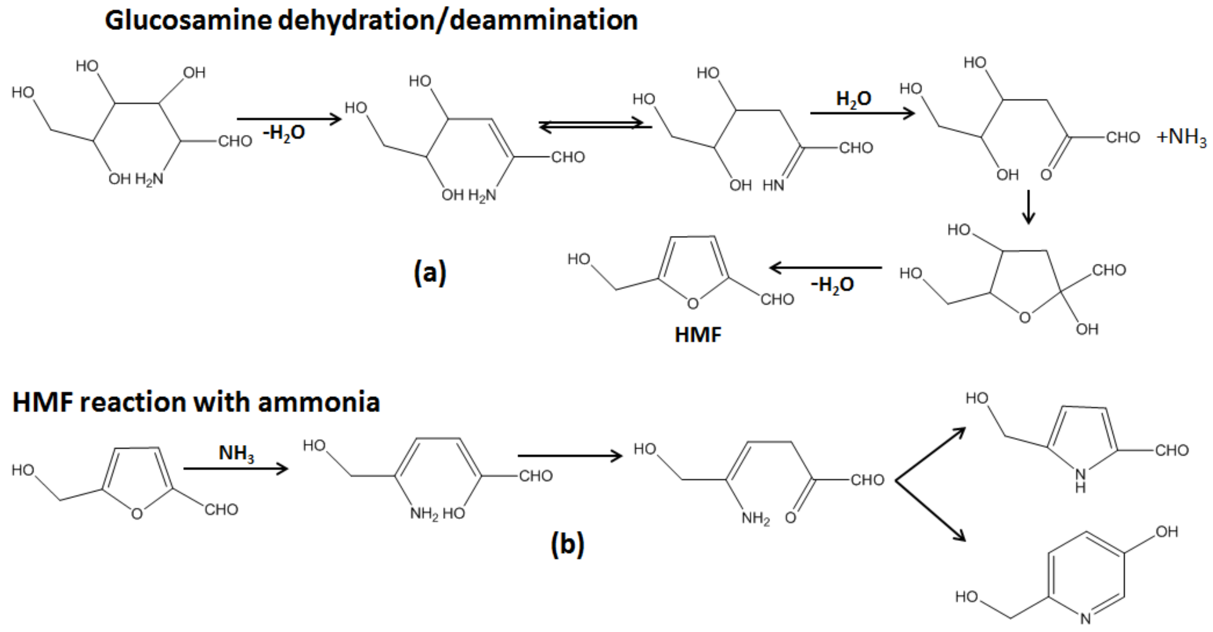

Scheme S2: Chemical reactions involved in: a) Glucose dehydration-deamination to HMF and ammonia; b) Reaction of HMF with ammonia.

References

1 Horvat, J., Klaić, B., Metelko, B. & Šunjić, V. Mechanism of levulinic acid formation. *Tetrahedron Letters* **26**, 2111-2114, doi:10.1016/s0040-4039(00)94793-2 (1985).

2 James, O. O. *et al.* Towards the conversion of carbohydrate biomass feedstocks to biofuels via hydroxylmethylfurfural. *Energy & Environmental Science* **3**, 1833-1850, doi:10.1039/b925869h (2010).

3 Chheda, J. N. & Dumesic, J. A. An overview of dehydration, aldol-condensation and hydrogenation processes for production of liquid alkanes from biomass-derived carbohydrates. *Catalysis Today* **123**, 59-70, doi:10.1016/j.cattod.2006.12.006 (2007).
